# Rapid fractionation of corn stover by microwave-assisted protic ionic liquid [TEA][HSO_4_] for fermentative acetone–butanol–ethanol production

**DOI:** 10.1186/s13068-024-02499-0

**Published:** 2024-05-07

**Authors:** Yankun Wang, Di Cai, Yongjie Jiang, Xueying Mei, Wenqiang Ren, Mingyuan Sun, Changsheng Su, Hui Cao, Changwei Zhang, Peiyong Qin

**Affiliations:** 1 National Energy R&D Center for Biorefinery, Beijing University of Chemical Technology, Beijing, 100029 People’s Republic of China; 2 College of Life Science and Technology, Beijing University of Chemical Technology, Beijing, 100029 People’s Republic of China; 3 Research Center for Eco-Environmental Sciences, Chinese Academy of Science, Beijing, 100085 China

**Keywords:** Corn stover, Protic ionic liquid, Microwave, Fractionation, Acetone–butanol–ethanol fermentation

## Abstract

**Background:**

The use of ionic liquids (ILs) to fractionate lignocelluloses for various bio-based chemicals productions is in the ascendant. On this basis, the protic ILs consisting of triethylammonium hydrogen sulfate ([TEA][HSO_4_]) possessed great promise due to the low price, low pollution, and high efficiency. In this study, the microwave-assistant [TEA][HSO_4_] fractionation process was established for corn stover fractionation, so as to facilitate the monomeric sugars production and supported the downstream acetone–butanol–ethanol (ABE) fermentation.

**Results:**

The assistance of microwave irradiation could obviously shorten the fractionation period of corn stover. Under the optimized condition (190 W for 3 min), high xylan removal (93.17 ± 0.63%) and delignification rate (72.90 ± 0.81%) were realized. The mechanisms for the promotion effect of the microwave to the protic ILs fractionation process were ascribed to the synergistic effect of the IL and microwaves to the depolymerization of lignocellulose through the ionic conduction, which can be clarified by the characterization of the pulps and the isolated lignin specimens. Downstream valorization of the fractionated pulps into ABE productions was also investigated. The [TEA][HSO_4_] free corn stover hydrolysate was capable of producing 12.58 g L^−1^ of ABE from overall 38.20 g L^−1^ of monomeric sugars without detoxification and additional nutrients supplementation.

**Conclusions:**

The assistance of microwave irradiation could significantly promote the corn stover fractionation by [TEA][HSO_4_]. Mass balance indicated that 8.1 g of ABE and 16.61 g of technical lignin can be generated from 100 g of raw corn stover based on the novel fractionation strategy.

**Supplementary Information:**

The online version contains supplementary material available at 10.1186/s13068-024-02499-0.

## Background

The increasing concerns of environmental pollutions and the unsustainable fossil chemicals supply make it urgent need to find sustainable alternative routes [1]. Lignocellulosic biomasses are mainly composed of polysaccharides in forms of cellulose and hemicellulose that able to be hydrolyzed into monomer sugars, as well as the amorphous polymeric lignin fractions that consist of series phenolic units, which have long been concerned as most abundant renewable resources substituted to the fossil oil for the production of various chemicals, fuels and materials [2]. Nevertheless, the direct valorization of the lignocelluloses is hindered by the inherent biomass recalcitrance, which is closely related to the morphological complexity of the cell walls and the heterogeneous nature [3]. A primary strategy for utilization of the lignocellulosic matrixes is taking a properly fractionation process to damage the stubborn structure of lignin and disrupt the crystalline of cellulose, so that obtaining a lignin predominated stream and a carbohydrate enriched pulp supported to various types of fermentations [4].

In the past decades, various biomass fractionation techniques, including physical, chemical, biological, and their combinations, have been developed [5–7]. Among them, ionic liquids (ILs) possessed good recyclability with nearly non-volatility, and recognized as a “green solvents” to reduce the biomass recalcitrance [8]. Generally, ILs that are commonly used for lignocellulose fractionation can be categorized into aprotic and protic ILs [9]. Although the aprotic ILs possessed high solubilizing properties of lignin and hemicellulose, shortcomings including the high cost of the precursors, the poor thermo-stability, the difficulties in recycling, and the low tolerance of humidity limited the realistic applications [10–12]. In contrast, the protic ILs that formed by proton exchange reaction of Brønsted base and acid precursors, were always much cheaper than the aprotic ILs [13]. For instance, the synthesis cost of triethylammonium hydrogen sulfate ([TEA][HSO_4_]), the typical protic IL, was only 1.24 $/kg, which was far lower than the 1-ethyl,3-methylimidazolium acetate ([EMIM][HSO_4_]) (20–101 $/kg), a kind of aprotic IL that was widely used in biomass fractionation [14]. Meanwhile, the protic ILs also possess high delignification capability [15].

The above features made the protic ILs great potential in biomass fractionation [15–17]. To date, various researches have been conducted on the decomposition of different types of lignocelluloses by the protic ILs. For instance, Chen et al. suggested to fractionate corn stover by [DBNH][Lev], and a reducing sugars yield of 0.80 g g^−1^ pulp was obtained [18]. Semerci et al. analyzed the capacities of various proton ionic liquids to the fractionation of lignocelluloses, among which, the highest glucose yield of 92% was obtained using [TEA][HSO_4_] [19]. Huang et al. suggested 80% of glucose can be recovered from poplar after [EOA][OAc] fractionation and the following enzymatic hydrolysis [20].

However, the current researches on the protic ILs fractionation of lignocelluloses were basically conducted by conventional energy-intensive heating progresses. As an alternative, the adoption of microwave irradiation enables the direct contacting between the biomass structure and the electromagnetic field, affording to an instantaneous temperature increase by volumetric heating [21]. Consequently, the lignocelluloses fractionation efficiency can be obviously improved [22]. Meanwhile, the thermal effect of microwave could promote the destroying of hydrogen bonds and increase the breakage of the crystalline arrangement of the cellulose fraction [23]. In literature, the synergistic effect of the ILs and microwave in the biomass fractionation processes have been proposed [24], which reflected the depolymerization of lignocellulose matrixes can be accelerated under milder conditions with obvious energy-reduction [25].

As aforementioned, the majority of previous researches on protonic ionic liquids were mainly focused on evaluating the pulps saccharification performances. However, there has been relatively limited assessment of the downstream valorization the saccharified pulps into biochemicals via fermentation. Herein, the current research addressed the microwave-assisted protic IL process for rapid fractionation of corn stover, aiming to effectively co-generation of technical lignins and the monomeric sugars that supported to downstream biobutanol production, a superior biofuel and bulk chemical, through acetone–butanol–ethanol (ABE) fermentation. The synergistic effect of the protic IL and microwave on the structural change and depolymerization of lignocellulose matrix was clarified. Based on the characterization results of the pulps and the isolated lignins, a comprehensive understanding of the mechanisms at microstructural and molecular level was realized. In addition, the inhibition effect of the residual [TEA][HSO_4_] in hydrolysate on the metabolism of the clostridia, the ABE producing strain was also highlighted, and the possibility for biobutanol production through the microwave-assistant [TEA][HSO_4_] fractionation route was investigated.

## Results

### Compositional analysis of the microwave-assistant [TEA][HSO4] fractionated corn stover pulp

Table 1 presents the chemical composition of corn stover pulps fractionated by [TEA][HSO_4_] under various conditions. Except for microwave heating, the conventional heating processes were treated as the references. The results indicate that the amorphous hemicellulose and lignin were easier hydrolyzed by [TEA][HSO_4_] no matter the heating strategies. The favorable removal of hemicellulose and delignification can be attributed to the anionic Brønsted acidity of [HSO_4_]^−^, acting as an acidic catalyst that cleaves the chemical bonds between lignin and cellulose while simultaneously dissolving lignin. Furthermore, due to the highly branched nature of hemicellulose, its glycosidic bonds degrade more rapidly under Brønsted acid catalysis [26]. In the control groups, with the increase of reaction time from 30 to 60 min, the xylan removal was increased from 82.19 ± 0.95% to 89.15 ± 0.88%. The extending of the reaction time from 30 to 90 min of the conventional heating processes also led to the lower glucan retention (from 75.04 ± 0.48% to 71.74 ± 0.71%). This is attributed to the dissolution effect of [TEA][HSO_4_] on cellulose, particularly the [HSO_4_]^−^ anion, which acts as a hydrogen bond acceptor for cellulose dissolution, disrupting both intra and intermolecular hydrogen bonds in cellulose molecules. This process leads to the generation of amorphous portions of cellulose, which dissolve under acidic conditions [27, 28].

**Table 1:** Compositional analysis of the pulps after fractionated by [TEA][HSO_4_] under microwave irradiation

Samples	Solid yield (wt%)	Chemical composition (wt%)			Glucan recovery (%)	Xylan removal (%)	Delignification (%)
		Glucan	Xylan	Lignin			
Untreated	–	38.20 ± 0.52	18.60 ± 0.20	24.80 ± 0.84	–	–	–
CHI-120 °C/30 min^a^	42.40 ± 1.35	65.13 ± 0.67	7.49 ± 0.57	19.67 ± 0.67	75.04 ± 0.48	82.82 ± 0.95	70.43 ± 0.77
CHI-120 °C /60 min	40.80 ± 1.26	66.93 ± 0.81	4.77 ± 0.82	17.06 ± 0.88	74.21 ± 0.68	89.15 ± 0.88	75.32 ± 0.87
CHI-120 °C /90 min	36.40 ± 1.57	72.53 ± 0.63	3.17 ± 0.91	23.15 ± 0.45	71.74 ± 0.71	93.61 ± 0.31	80.44 ± 0.67
MI-130 W/3 min^b^	53.04 ± 0.92	52.88 ± 0.55	10.94 ± 0.33	21.58 ± 0.31	76.21 ± 0.56	67.64 ± 0.55	59.41 ± 0.85
MI-150 W/3 min	49.39 ± 1.02	57.36 ± 0.71	7.83 ± 0.55	19.33 ± 0.42	76.97 ± 0.68	78.42 ± 0.75	66.15 ± 0.67
MI-170 W/3 min	47.06 ± 0.88	59.86 ± 0.89	5.23 ± 0.74	19.35 ± 0.96	76.54 ± 0.49	86.27 ± 0.34	67.71 ± 0.74
MI-190 W/3 min	43.10 ± 1.41	65.84 ± 1.02	2.84 ± 0.76	17.73 ± 0.65	77.11 ± 0.77	93.17 ± 0.63	72.90 ± 0.81
MI-210 W/3 min	38.42 ± 0.89	72.22 ± 0.84	2.15 ± 0.56	20.60 ± 0.47	75.39 ± 0.66	95.39 ± 0.72	71.93 ± 0.61
MI-230 W/3 min	36.63 ± 1.06	73.69 ± 0.43	1.66 ± 0.22	22.31 ± 0.36	73.35 ± 0.83	96.62 ± 0.65	71.01 ± 0.82

^a^CHI: conventional heating for IL fractionation

^b^MI: microwave heating for IL fractionation

By contrast, because of the obvious shortening of the pulping time in the microwave-assistant groups (only 3 min for heating), higher glucan retention can be realized accompanied by the more rapid xylan hydrolysis and delignification. For instance, 77.11 ± 0.77% glucan can be recovered in the MI-190 W/3 min pulp, which was 5.37% higher than that of the conventional heating process with similar terminal temperature (detailed heating curves for various microwave powers during the fractionation are shown in Additional file 1: Figure S1). Therefore, by the assistance of microwave irradiation, temperature and the duration for the corn stover fractionation can be significantly reduced. These superiorities could be attributed to the directly disruption of the biomass cell wall structure by enhanced solvent effect [29]. Furthermore, based on the ionic properties of [TEA][HSO4], ILs exhibit enhanced absorption of microwave irradiation. Microwave radiation significantly alleviates the mass transfer resistance caused by the high viscosity of [TEA][HSO_4_] and the biomass structure, facilitating the penetration of [TEA][HSO_4_] into the biomass and improving the degradation of lignocellulosic substrates [30, 31].

Results in Table 1 also illustrated the corn stover fractionation efficiency depended on the power of microwave. As the microwave power increased from 130 to 190 W, the xylan hydrolysis rate was increased from 67.64 ± 0.55 to 93.17 ± 0.63%. Meanwhile, the delignification was increased from 59.41 ± 0.85 to 72.90 ± 0.81%. However, the delignification and xylan removal were almost remaining in constant when the microwave power higher than 190 W. At the same time, the glucan recovery was decreased than the lower microwave power assisted groups. This phenomenon can be ascribed to the higher terminal temperatures for corn stover fractionation. The higher temperature in protic ILs fractionation would result in non-lignin components formation. For instance, literature has been pointed out that the dissolution and dehydration of xylan in lignocellulose can be promoted by the acidic ILs at high temperatures [32]. Consequently, the intermediates such as 5-HMF and furfural were undergone condensation, and finally forming the pseudolignin (as humus) [33], and the coagulated higher molecular weight lignin as well as pseudolignin were deposited on the surface of pulp [15]. Literature also suggested the formation of pseudolignin during the fractionation process would further negatively influence on the enzymatic saccharification of the pulp [34]. This statement can be proved by Fig. 4.

### Characterization and the enzymatic fractionation of pulps

**Fig. 1:**
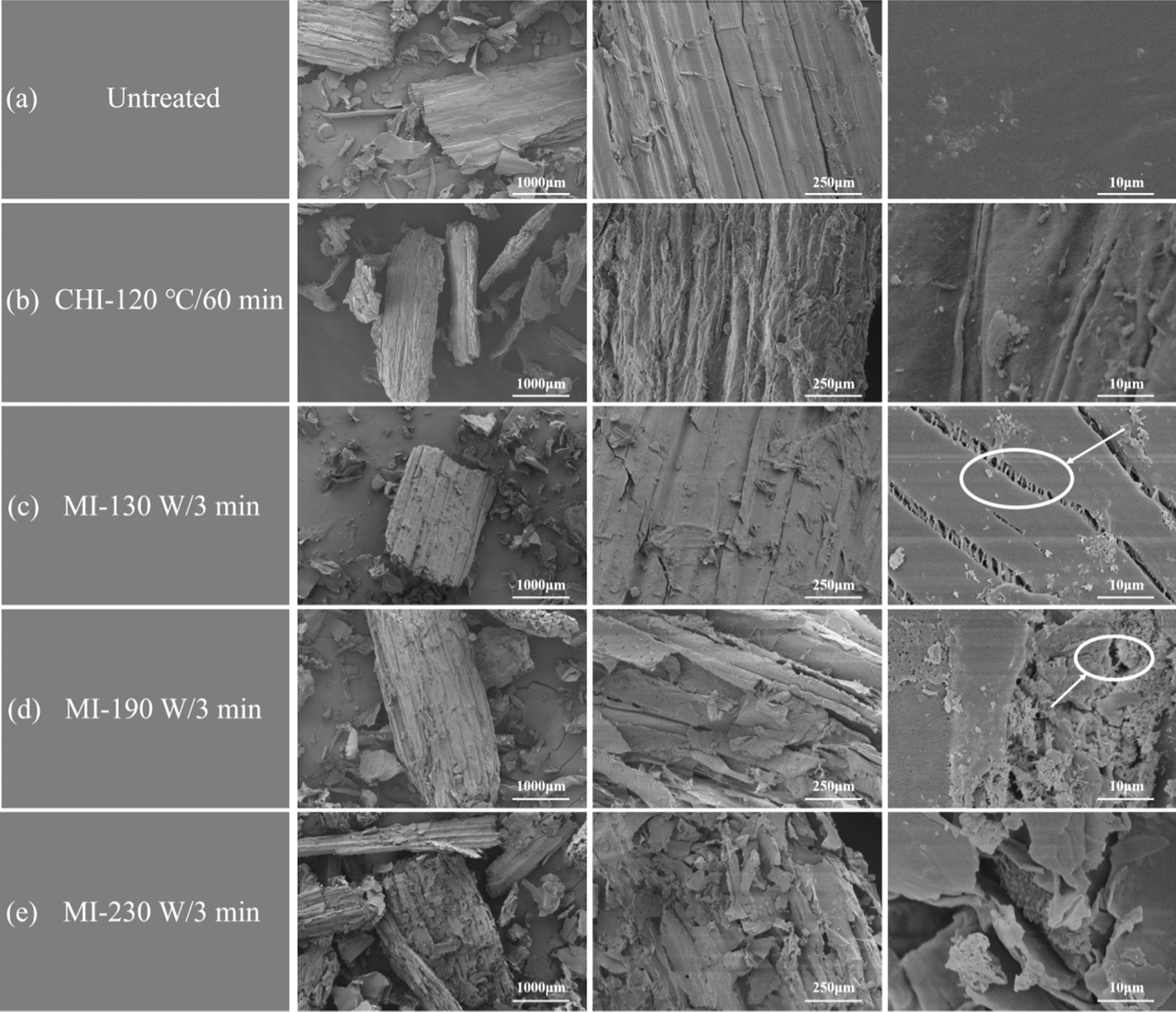
SEM images of the microwave-assistant [TEA][HSO_4_] fractionated corn stover. **a** Untreated corn stover; **b** pulps obtained by the conventional heating; **c**–**e** pulps obtained by the microwave heating under different conditions

To further reveal the synergistic effect of the microwave and [TEA][HSO_4_] in fractionation process, the physical and chemical properties of the pulp were characterized. Generally, the surface morphology of the untreated corn stover showed intact and rigid microfibrillar structure (Fig. 1). After [TEA][HSO_4_] fractionation, the surface of pulp was disrupted and forming irregular appearance. Compared with the conventional heating groups, the pulps obtained in microwave-assisted groups exhibited rougher surfaces with deeper grooves, inferring the more efficiently disruption of the chemical structure of corn stover.

**Fig. 2:**
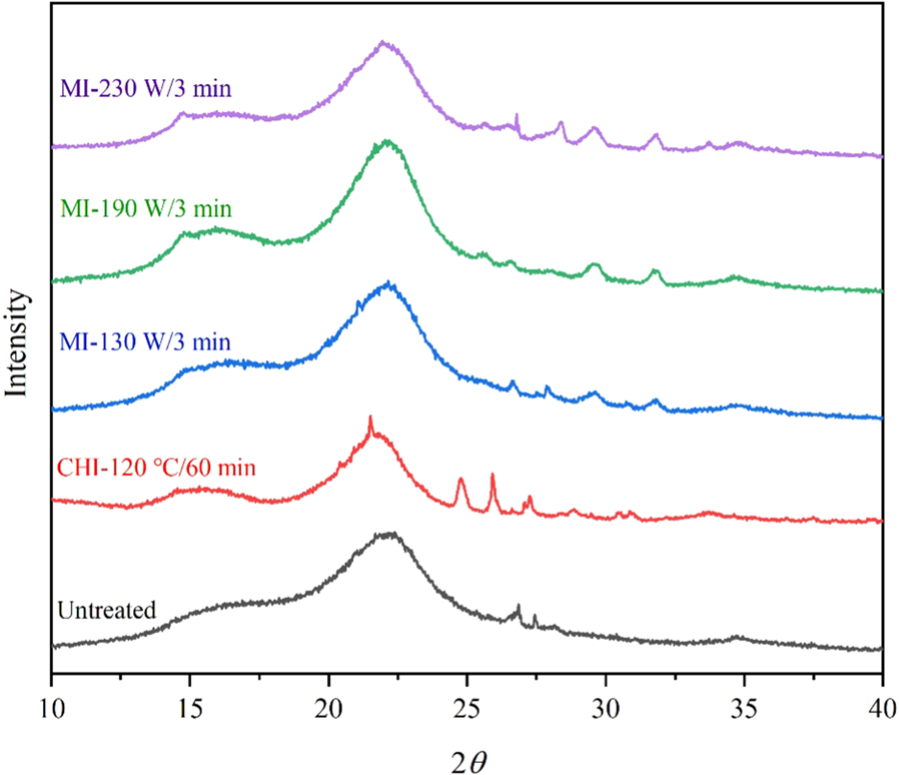
X-ray diffraction (XRD) patterns of corn stover pulps fractionated by [TEA][HSO_4_] under different conditions

As shown in Table 2, the XRD pattern indicated the CrI of the pulp obtained by 120 °C of conventional heating (52.03%) was little lower than the MI-190 W/3 min group (57.17%) that with similar terminated temperature, confirming the more obvious cracking of the pulp surface by the effect microwave irradiation [35]. Additionally, as the increase of the microwave power in IL fractionation, the CrI of the pulps were also increased. This can be ascribed to the efficient cleavage and dissolution of amorphous regions in pulps [28, 36]. However, CrI of the pulp in the MI-230 W/3 min group was only 54.72%. In this group, the intensity at around 16.0° was dropped, and the broad peak becoming weak shoulder, indicating the severely distortion of cellulose I lattice (Fig. 2) [37].

**Table 2:** CrI of the untreated and the [TEA][HSO_4_] fractionated corn stover pulps

Specimens	CrI (%)
Untreated	44.96
CHI-120 ℃/60 min	56.47
MI-130 W/3 min	49.74
MI-190 W/3 min	57.17
MI-230 W/3 min	54.72

**Fig. 3:**
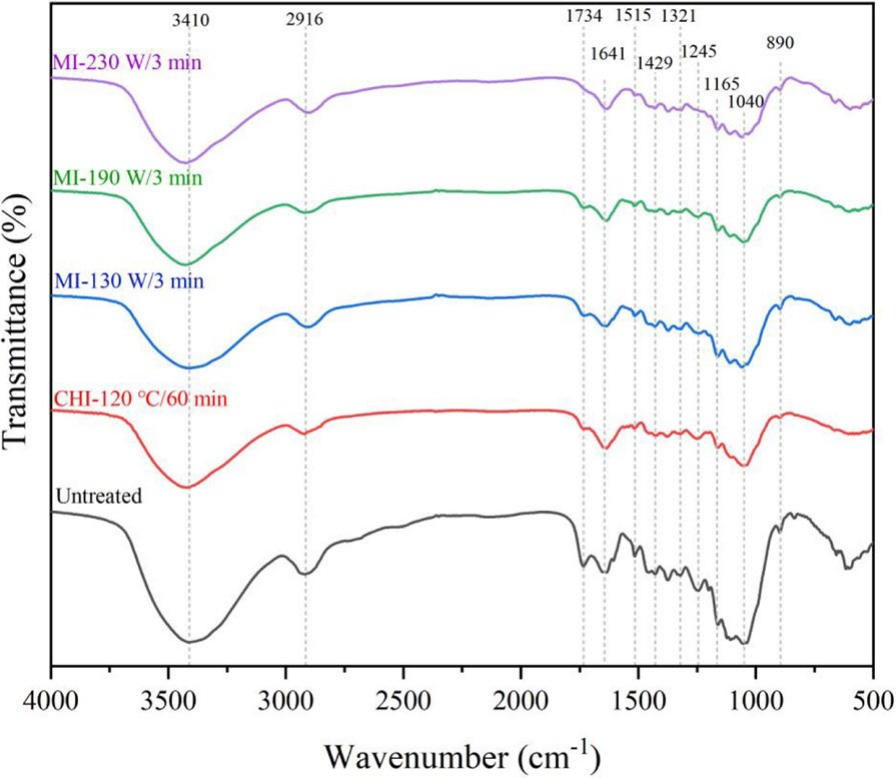
FT-IR spectrums of the microwave-assistant [TEA][HSO_4_] fractionated corn stover pulps

FT-IR spectrums illustrated the weakening of the absorption peak at 3410 cm^−1^ in the [TEA][HSO_4_] fractionated pulps in contrast to the untreated corn stover, owing to the reduction of free hydroxyl groups after substantial hemicellulose and lignin removal by the protic IL fractionation (Fig. 3) [38]. Furthermore, the absorption peaks at 1515 cm^−1^ (attributed to the aromatic skeletal vibration) and 1734 cm^−1^ (attributed to the unconjugated C=O in hemicellulose) were gradually weakened with the increase of microwave power, indicating the excessive delignification and hemicellulose removal under harsher conditions [28, 39]. Besides, the pulps obtained from the CHI-120 ℃/60 min and MI-190 W/3 min groups exhibit obvious weakening trend at 890 cm^−1^ compared to the untreated corn stover, representing the removal of the amorphous components. In contrast, the absorption peak at 890 cm^−1^ in the pulp fractionated by MI-230 W/3 min was enhanced, signifying the transformation of cellulose I to the amorphous form [35]. As it is illustrated in literature, attribute to the broken down of the intramolecular hydrogen bonds by protic ILs, especially [HSO_4_]^−^ anion acts as a hydrogen bond acceptor for cellulose dissolution, cellulose which regenerates after pretreatment tends to be more amorphous [27]. All these statements coincided with the results shown in Table 2.

The aforementioned characterization results of the microwave-assisted [TEA][HSO_4_] fractionated pulps demonstrate the enhanced removal of hemicellulose and delignification compared with the conventional heating. The effective depolymerization of the recalcitrance lignocellulosic matrix structure and the exposure of the cellulose structure would promote the enzymatic saccharification for fermentable monomeric sugars production [19].

**Fig. 4:**
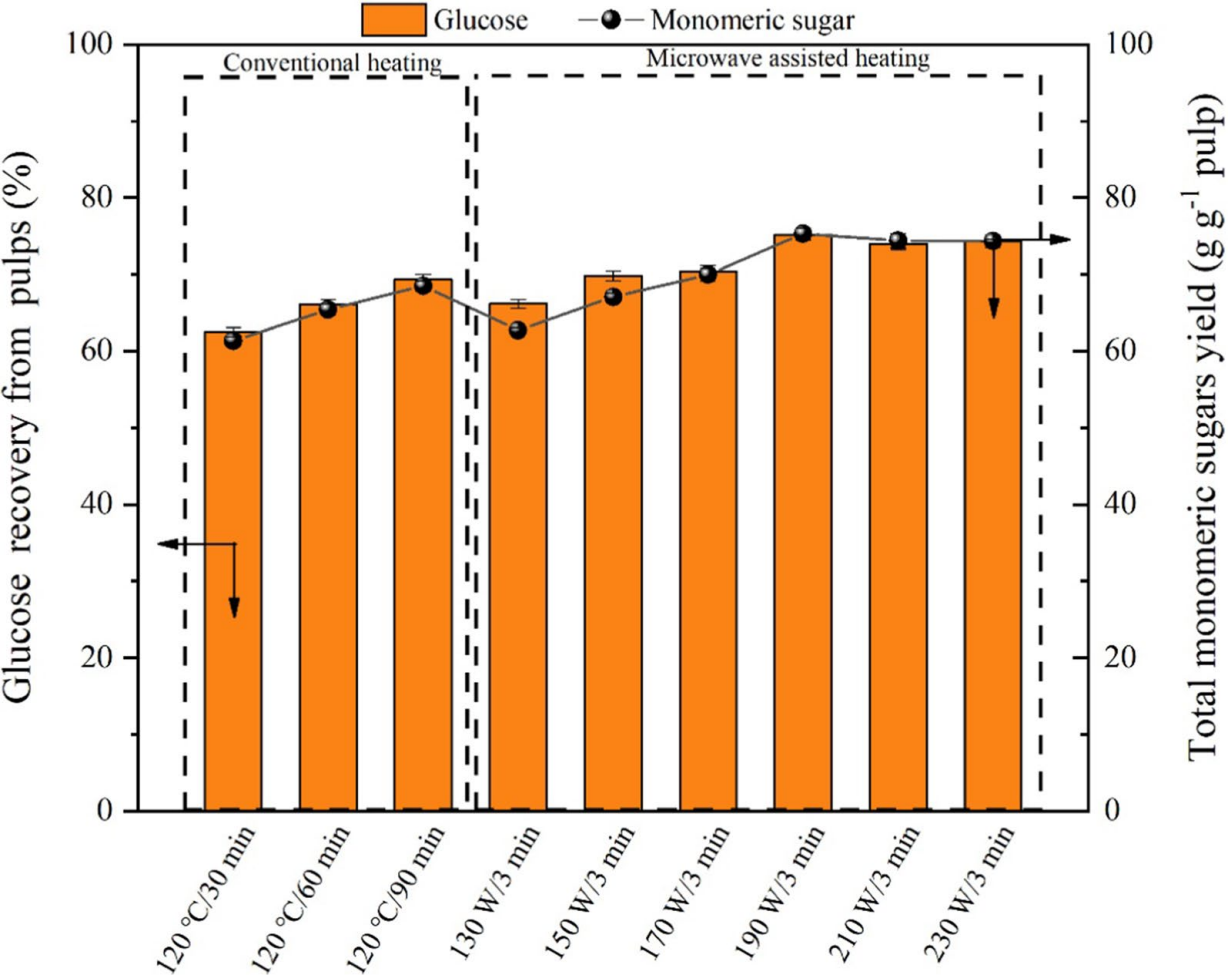
The monomeric sugar yields by enzymatic hydrolysis of the corn stover pulps

**Fig. 5:**
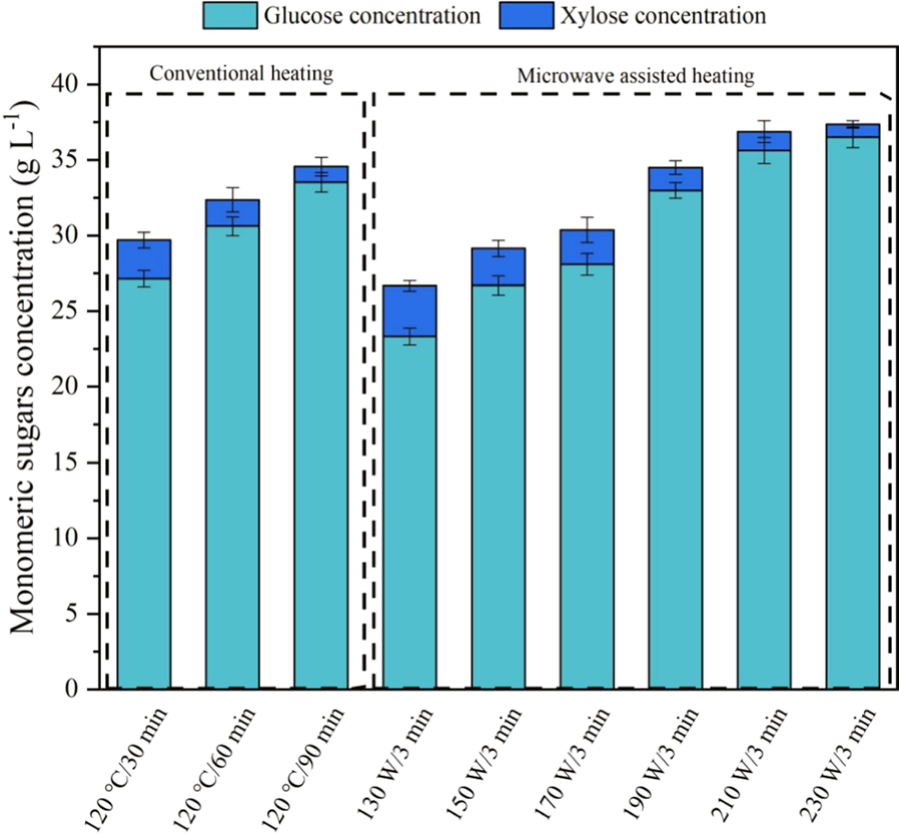
Monomeric sugars concentrations in the enzymatic hydrolysates of the corn stover pulps

The enzymatic hydrolysis performances of the fractionated pulps were further investigated. The enzymatic hydrolysis efficiency of the pulps was highly dependent on the delignification and xylan removal from the corn stover feedstock. (The correlations between delignification and cellulose content with the glucose yield are shown in Additional file 1: Figures S2 and S3.) As expected, the monomeric sugars production from the pulps of the conventional heating groups were behind the microwave-assisted groups, though longer reaction time was adopted (Fig. 4). The highest monomeric sugars recovery (75.32 ± 0.67% from pulp) can be realized in the MI-190 W/3 min among the tested groups. Glucose and xylose concentrations of 32.98 ± 0.52 g L^−1^ and 1.51 ± 0.45 g L^−1^ (Fig. 5), respectively, can be realized when the pulp dosage rate was 6% (w/v). However, as expected, because of the formation of pseudolignin in higher microwave power groups (> 210 W), the hydrolysis rate of the pulps was lower than that of the milder conditions. Current advances in glucose yield from lignocelluloses by various protic ILs fractionations indicated similar glucose production can be realized under lower temperature and shorter reaction time with the assistance of microwave heating in the current work (Additional file 1: Table S1).

### Characterization of the isolated lignin specimens

**Fig. 6:**
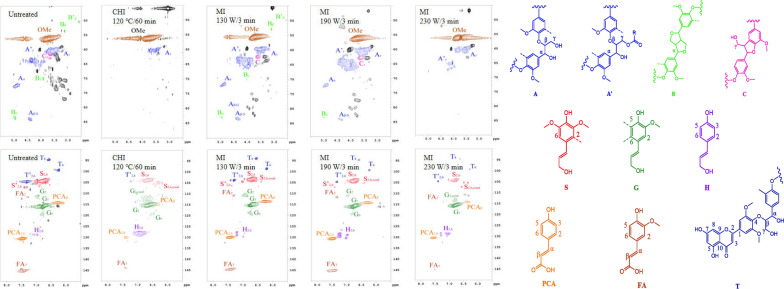
HSQC NMR spectrums of the lignin specimens after [TEA][HSO_4_] fractionation by various conditions

In order to verify the positive effect of microwave on the delignification of corn stover during the [TEA][HSO_4_] fractionation, and clarify the characterizations of the isolated lignin streams for further valorization, the subunit composition, degree of condensation, and the presence of connecting bonds in the isolated lignin were analyzed by 2D HSQC NMR spectroscopy. As illustrated in Fig. 6, various subunits and connecting bonds in native lignin were trend to change by [TEA][HSO_4_] fractionation no matter the heating process selected (signal assignments in spectrums are listed in Additional file 1: Table S2). The semi-quantitative results regarding the units and connecting bonds in lignin structure are shown in Table 3, which indicated that the effective delignification of corn stover in the [TEA][HSO_4_] fractionation process was primarily ascribed to the promotion of lignin dissolution through the cleavage of β-*O*-4ʹ [33]. As it was indicated in literature, the anion in [TEA][HSO_4_] acted as a nucleophile and attacks electron-deficient sites in the lignin structure, resulted in the effective cleavage of the β-*O*-4ʹ bonds [40]. Compared with the native lignin of corn stover with β-*O*-4ʹ content of 56.06%, the β-*O*-4ʹ bond in the isolated lignin by [TEA][HSO_4_] fractionation with conventional heating was only 13.35%, while it was further decreased to 12.97% in the lignin specimen in the MI-230 W/3 min group. Meanwhile, the more stable C–C bonds in lignin, including the β-5ʹ and β-βʹ, were also obvious decreased after [TEA][HSO_4_] fractionation.

**Table 3:** Semi-quantitative information of lignin functionalities according to HSQC NMR

Specimens	Aromatics (%)			Linkages (per 100 of C_9_ units^a^)			*S*/*G*
	*S*	*G*	*H*	β-*O*-4	β-β	β-5	
Untreated	64.18	30.81	5.01	56.06	7.87	5.18	1.04
CHI-120 ℃/60 min	37.52	35.87	26.60	13.55	1.89	1.35	0.52
MI-130 W/3 min	50.82	28.25	20.93	26.34	6.55	2.25	0.92
MI-190 W/3 min	45.93	27.05	27.02	18.21	4.29	1.92	0.85
MI-230 W/3 min	30.26	44.37	25.37	12.97	1.28	0.80	0.68

^a^C_9_ is calculated from HSQC NMR spectra signals by 0.5 IS_2,6_ + IG_2_ + 0.5 IH_2, 6_

**Scheme 1:**
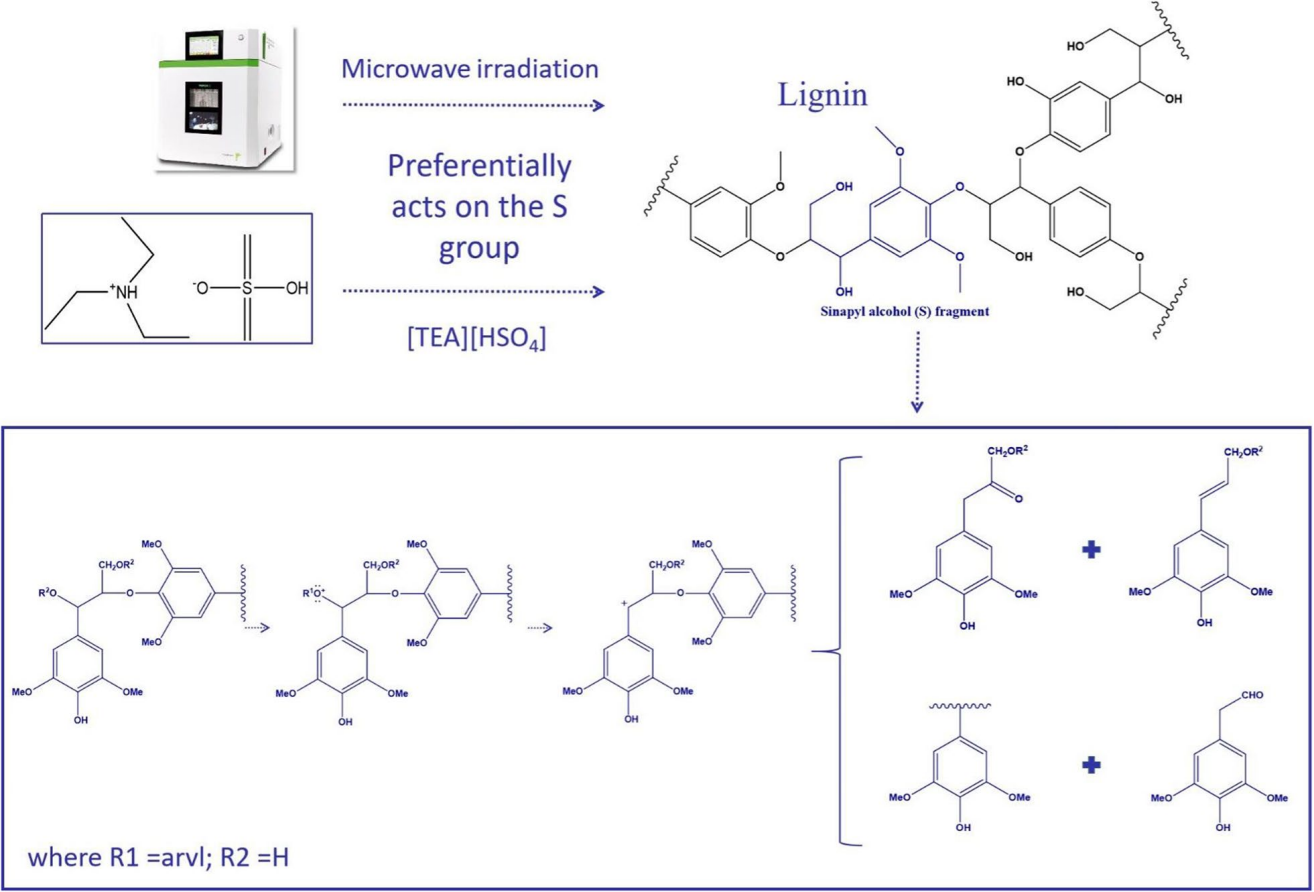
Proposed pathway for the depolymerization of S units in lignin by microwave-assistant [TEA][HSO_4_]

From the spectrum of the aromatic region, it can be found that with the increase of microwave power, *S*_2, cond._ and *G*_2, cond._ signals were enhanced, indicating that the condensation occurred on the syringyl (*S*) and guaiacyl (*G*) during the [TEA][HSO_4_] fractionation. Meanwhile, an enhanced *p*-hydroxyphenyl (*H*) signal was also observed, suggesting the conversion of *p*-coumaric acid (PCA) to H-type polymers in the fractionation process [33]. The semi-quantitative information in Table 3 shows that an increase in microwave power leads to decreases of the *S*/*G* value. In contrast to the *S*/*G* of 1.04 in untreated corn stover, this value was gradually decreased from 0.92 in the MI-130 W/3 min group to 0.68 in the MI-230 W/3 min group. This phenomenon can be explained by the more reactive aryl ether bonds of the S unit in acidic IL media [25]. Varanasi et al. reported lignin undergoes selective degradation in aqueous ionic liquids depending on temperature, with the *S* units preferentially decomposing at high temperatures, while the *G* units more prone to depolymerization at low temperatures. This degradation mechanism is similar to the degradation mechanisms of lignin under acidic and alkaline conditions [41]. Regarding the experimental results of this study, the depolymerization mechanism of lignin in synergy with microwave and [TEA][HSO_4_] appears to be more similar to the acidic degradation mechanism of lignin (Scheme 1).

**Fig. 7:**
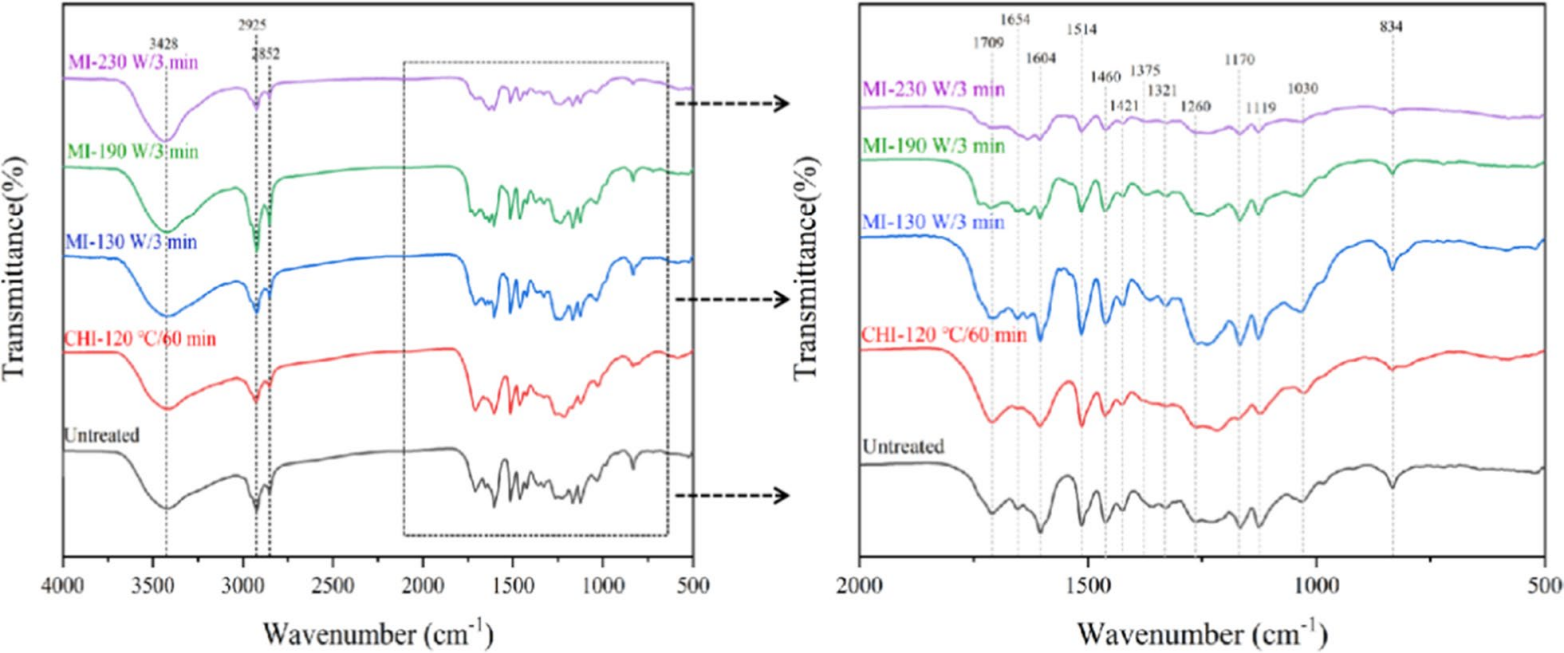
FT-IR spectrums of the isolated lignin specimens

To characterize in detail the functional groups and clarify the cleavage of the interlinkages of lignin during the microwave-assistant [TEA][HSO_4_] fractionation, FT-IR was further conducted and the spectrums are shown in Fig. 7. The absorption peaks at 1604 cm^−1^, 1514 cm^−1^, 1421 cm^−1^, and 1119 cm^−1^ are attributed to aromatic ring vibrations and methyl deformations in lignin specimens [42]. The absorption peaks at 1460 cm^−1^ and 1375 cm^−1^ are attributed to C–H and O–H bending vibrations that come from aliphatic and aromatic hydroxyl groups [40]. Besides, the absorption peak at 1321 cm^−1^ corresponds to the stretching vibration of the *S* unit, while the peak at 1260 cm^−1^ corresponds to the *G* unit [35]. The signal for absorption peak at 1260 cm^−1^ and 1321 cm^−1^ gradually decreased with the increase of the microwave power, which is also consistent with the statement for the decomposition of the *S* and *G* units in Fig. 7. Meanwhile, it is also worthy to be noted here that the absorption peak at 1030 cm^−1^ was gradually disappearance accompany with the increase of the microwave power. Therefore, the C–OH and C–O–C linkages in the side chains and glycosidic bonds can be effectively cleaved by microwave heating process [43], inferring the decisive role of the microwave irradiation to the depolymerization of the lignin–carbohydrate complexes (LCC) [24]. Since the presence of LCC would negatively influenced on the delignification, hemicellulose removal, and the enzymatic hydrolysis of the carbohydrates [44], it might be another reason for the boosting monomeric sugars production in the microwave-assistant groups.

The molecular weight of the lignin specimens was analyzed by GPC (Table 4). *M*_w_ and *M*_n_ of the isolated lignin after [TEA][HSO_4_] fractionation became lower, attributed to the cleavage of the intermolecular bonds in native lignin. Nevertheless, in comparison with the little changed *Mn*, the *Mw* of the lignin specimens isolated from the microwave heating groups were higher than the conventional heating process. Correspondingly, the PDI of the lignin in microwave groups were also higher than the conventional heating groups that with narrow molecular weight distribution of the isolated lignin.

**Table 4:** Molar mass of the isolated lignin specimens

Specimens	*Mw* (g mol^−1^)	*Mn* (g mol^−1^)	PDI
Untreated	4038	1205	3.31
CHI-120 ℃/60 min	858	629	1.39
MI-130 W/3 min	1263	689	1.83
MI-190 W/3 min	1165	657	1.76
MI-230 W/3 min	1140	639	1.78

### ABE fermentation using the enzymatic hydrolysate

The enzymatic hydrolysate of the corn stover pulp fractionated by microwave-assistant [TEA][HSO_4_] was attempted to valorize into biobutanol, the advanced biofuel, by ABE fermentation of *Clostridia* sp. [45]. Herein, a hyper butanol production *C. acetobutylicum* ABE-P 1201 strain that possessed high robustness and phenolic compounds tolerance in lignocellulose hydrolysates was adopted [46]. Although there were numerous researches have been down focused on the effective depolymerization of the lignocellulose matrixes via protic ILs for low-price monomeric sugars production [20, 33, 47], the downstream fermentative transformations of the corresponding hydrolysates were still rarely reported.

**Fig. 8:**
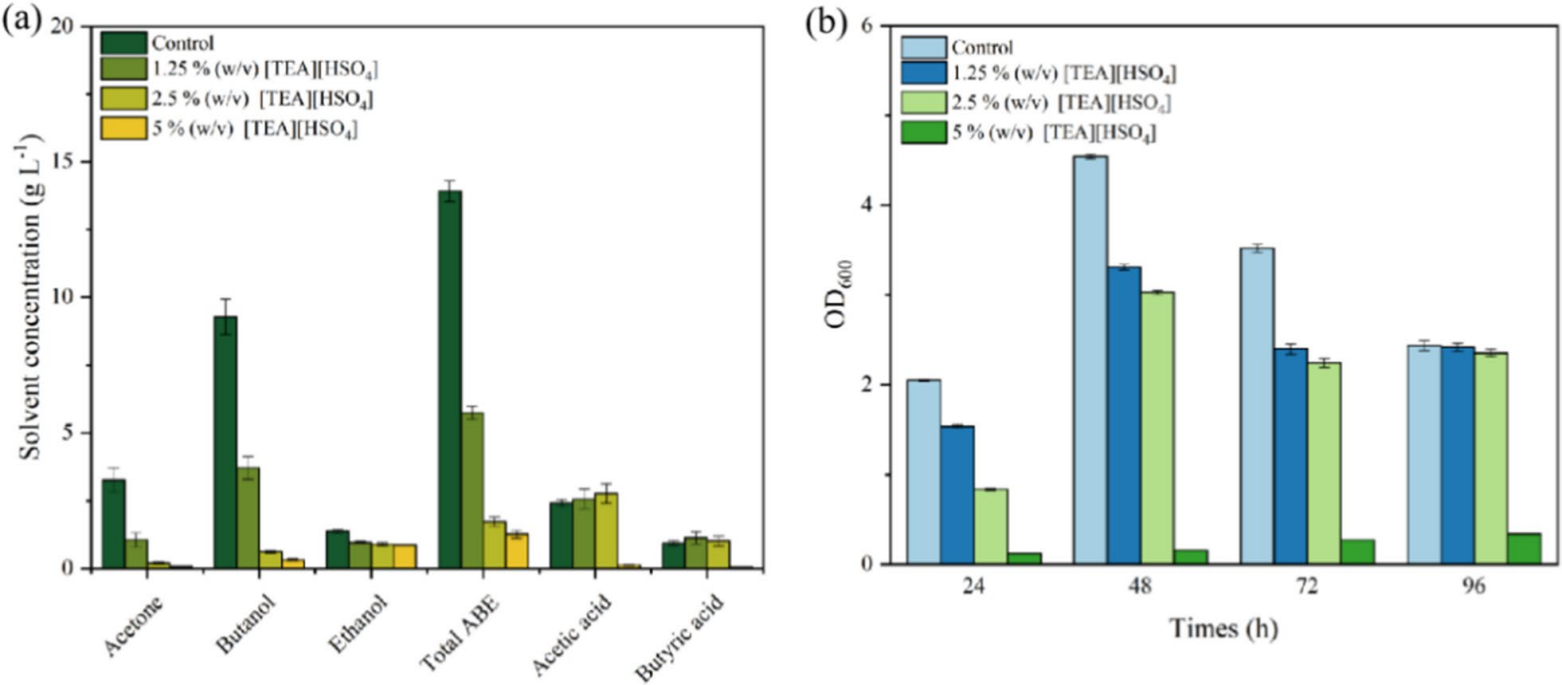
The inhibition effects of [TEA][HSO_4_] on ABE production by *Clostridium acetobutylicum* ABE P-1201. **a** Solvents concentration in the end fermentation broth, and **b** changes of OD_600_ during batch fermentation. The synthetic medium that containing the similar constitution of the monomeric sugars in the corn stover hydrolysate was used as substrate

In previous works, it was suggested microbes in IL aqueous solution could have suffered from activity loss due to the higher salt concentration and ionic strength [48]. Thus, before carrying out the ABE fermentation using the realistic corn stover hydrolysate, the inhibition of the residual ILs that was dissolved in the enzymatic hydrolysate to the cell’s growth and the metabolism of the ABE solvents production was investigated. As illustrated in Fig. 8, the protic [TEA][HSO_4_] IL exhibited inhibition to both of the clostridia cells’ growth and the ABE production in batch fermentation process. In contrast to the 2.43 of OD_600_ in the control group without addition of IL in substrate, only 0.37 of OD_600_ was detected in the final fermentation broth when using the initial substrate that containing 5 wt% of [TEA][HSO_4_]. Therefore, the cells growth was almost shutting down because of the toxic effect of the high concentrate protic IL. Meanwhile, the ABE concentration was gradually decrease to 1.27 ± 0.13 g L^−1^ in the 5 wt% of [TEA][HSO_4_] containing group from 13.91 ± 0.44 g L^−1^ in the control group without IL. Organic acids by-product, however, was slightly increased with the increase of [TEA][HSO_4_] concentration until the IL in initial substrate was higher than 2.5 wt%, suggesting the inhibition of the protic IL to the metabolism of carbon source by clostridia was severer in the solventogenesis phase than the acidogenesis phase [49]. Hence, unlike the relatively better biocompatibility of the choline- or imidazole-based ILs, the [TEA][HSO_4_] exhibited more obvious inhibition to the metabolism of the microorganisms.

After expanding the scale of enzymatic hydrolysis to 500 mL in conical flask, an overall 38.23 ± 0.48 g L^−1^ of monomeric sugars (including 36.17 ± 0.34 g L^−1^ of glucose and 2.06 ± 0.14 g L^−1^ of xylose) was detected in hydrolysate of the pulp from the MI-230 W/3 min group (the solid loading was 6%, w/v), which was similar to the results in Fig. 4. In addition, the residual [TEA][HSO_4_] concentration in the hydrolysate was 0.15 ± 0.01 g L^−1^, which was far lower than the tested group in Fig. 8.

**Fig. 9:**
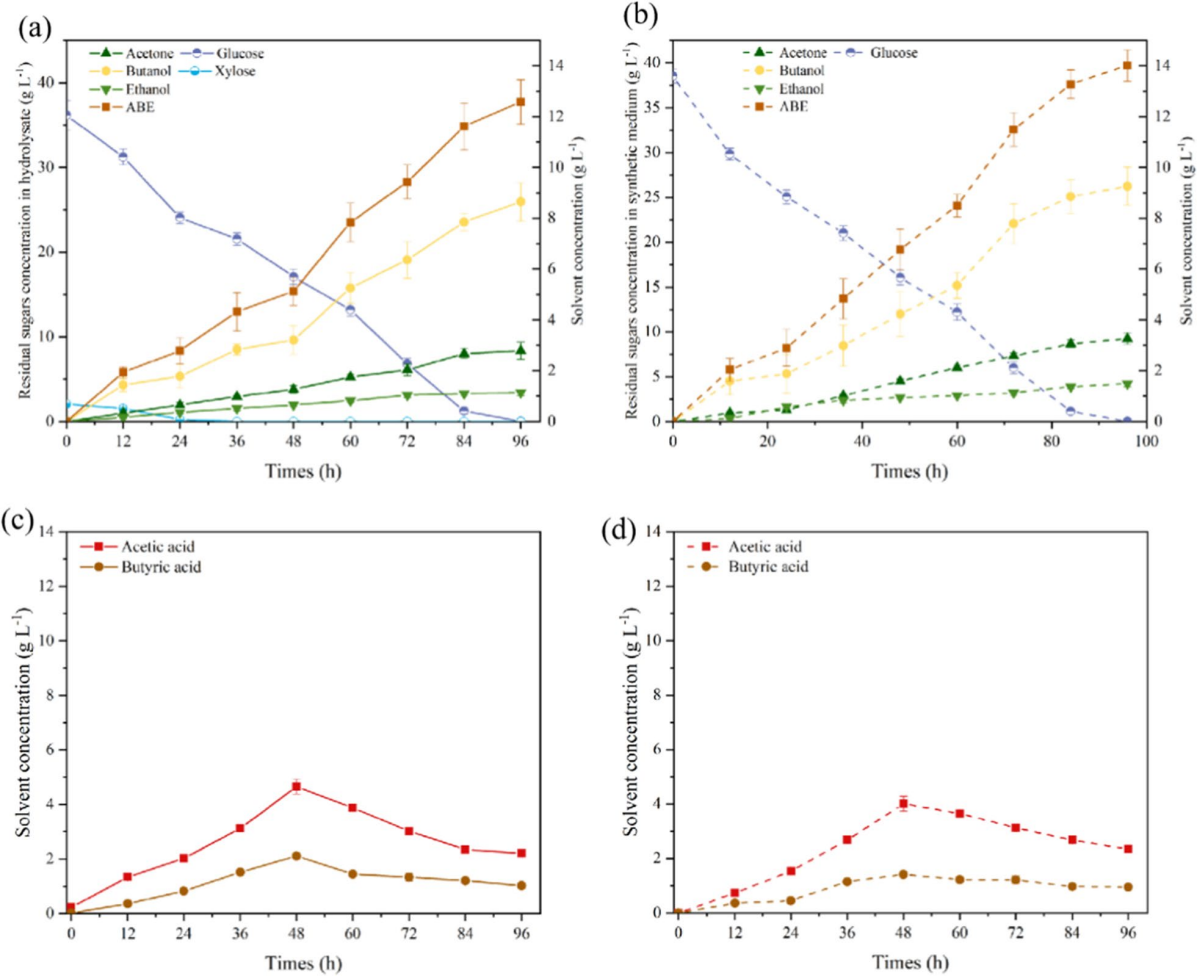
ABE fermentation using the enzymatic hydrolysate of the microwave-assistant [TEA][HSO_4_] fractionated pulp. Time course of the solvents production and residual sugars concentration using **a** hydrolysates and **b** synthetic medium; and organic acids concentration using **c** hydrolysates and **d** synthetic medium

**Fig. 10:**
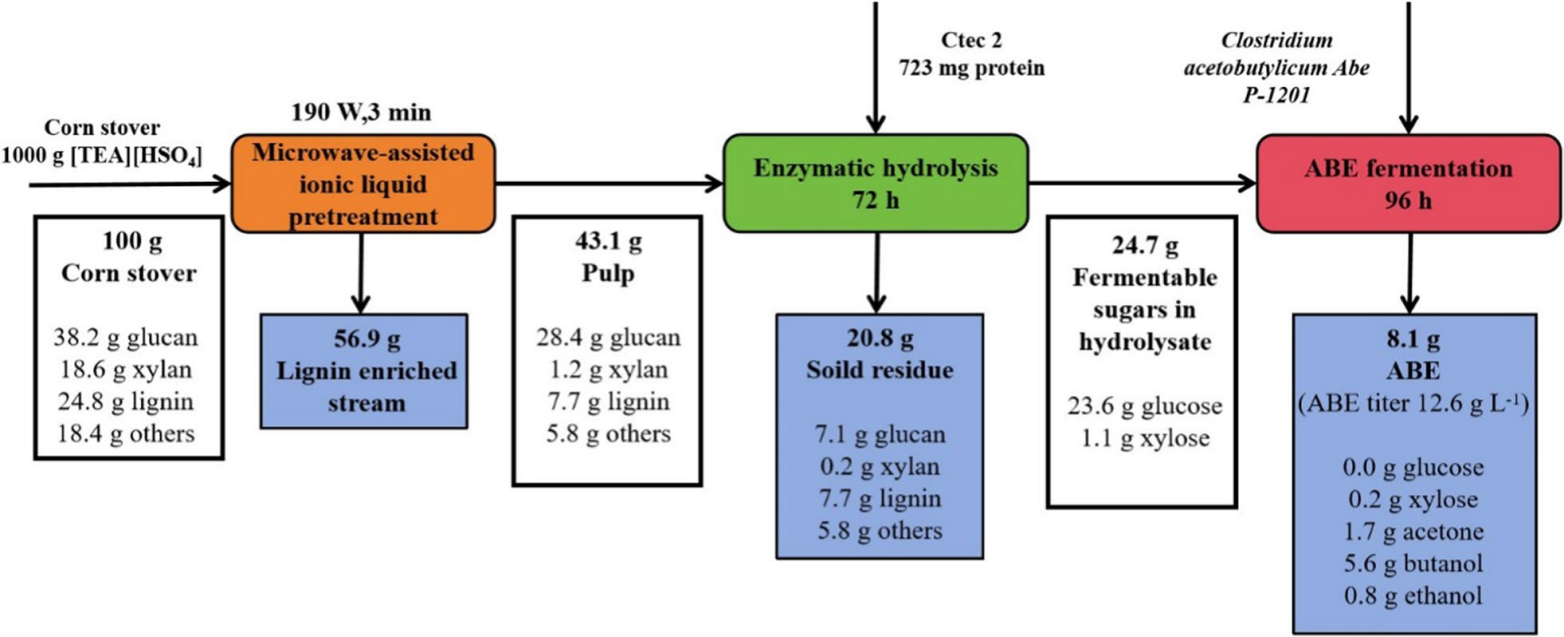
Mass balance of ABE production from corn stover based on the microwave-assistant [TEA][HSO_4_] fractionation process

Time course for the batch ABE fermentation using the enzymatic hydrolysate of the pulp from MI-190 W/3 min group is shown in Fig. 9. As expected, the corn stover hydrolysate capable of being used as the substrate for ABE solvents production without detoxification and nutrients supplementation. After 96 h of inoculation, 12.58 ± 1.29 g L^−1^ of ABE (including 2.79 ± 0.34 g L^−1^ of acetone, 8.65 ± 0.75 g L^−1^ of butanol, and 1.13 ± 0.12 g L^−1^ of ethanol) were detected in the fermentation broth, which was slightly behind to the synthetic medium without IL (Table 5). Mass balance of the ABE productions from corn stover based on the microwave-assistant [TEA][HSO_4_] fractionation process is shown in Fig. 10. About 8.1 g of ABE production can be yielded from 100 g of corn stover. At the same time, 16.61 g of technical lignin could be co-generated from the liquid phase after IL recycling.

**Table 5:** Key parameters of the batch ABE fermentation using the corn stover hydrolysate based on [TEA][HSO_4_] fractionation

Parameters	Hydrolysates	Synthetic medium
Solvent conc. (g L^−1^)		
Acetone	2.79 ± 0.34	3.26 ± 0.22
Butanol	8.65 ± 0.75	9.26 ± 0.75
Ethanol	1.14 ± 0.12	1.49 ± 0.12
Total ABE	12.58 ± 1.29	14.01 ± 1.09
ABE yield (g g^−1^)		
Acetone	0.07 ± 0.00	0.08 ± 0.00
Butanol	0.23 ± 0.01	0.24 ± 0.01
Ethanol	0.03 ± 0.00	0.04 ± 0.00
Total ABE	0.33 ± 0.01	0.36 ± 0.01
ABE productivity (g L^−1^ h^−1^)	0.13 ± 0.01	0.14 ± 0.01
Acids conc. (g L^−1^)		
Acetic acid	2.21 ± 0.12	2.42 ± 0.15
Butyric acid	1.03 ± 0.04	0.94 ± 0.06
Fermentation period (h)	96	96

## Conclusions

In this study, microwave-assistant [TEA][HSO_4_] fractionation process was established and proved as an effective method that was capable of co-generation of biological ABE and technical lignin co-generation. The synergistic effect of the microwave irradiation with the IL to the depolymerization of the highly recalcitrance lignocellulose matrixes was clarified by characterization of the pulps and the isolated lignin specimens. Compared with the conventional heating process, the microwave-assistant protic IL fractionation exhibited superiorities in lower energy requirement and higher efficiency. Moreover, the enzymatic hydrolysate of the fractionated pulps can be used as the substrate for valuable ABE solvents production without detoxification and additional nutrients. Therefore, the microwave-assistant [TEA][HSO_4_] fractionation showed great promising in effective and cleaner valorization of the low-valuable lignocelluloses into renewable chemicals and fuels.

## Method and materials

### Raw material

Corn stover was harvested in Suihua, Heilongjiang Province, China. After drying out and milling into 20–60 meshes, the corn stover powder was stored at -20 °C before use. Cellulase (121.2 ± 1.3 FPU mL^−1^, 130.5 ± 2.3 mg mL^−1^ protein) was obtained from Novozymes. All the chemicals were purchased from Macklin Biochemical Co., Ltd.

### Ionic liquid fractionation of corn stover

The protic IL [TEA][HSO_4_] was synthesized generally according to the protocol in literature with little modification [50]. The detail methods as follow: 5 M of H_2_SO_4_ was mixed with triethylamine (≥ 99 wt%) in a low-temperature water bath (− 5 °C) under molar ratio of 1:1, and stirred for 30 min. Moisture in [TEA][HSO_4_] was reduced through vacuum evaporation, and subsequent determination using a Fischer titration instrument revealed a water content of 20 wt%, aiming to reduce the viscosity and the overall cost of the ILs, [50].

For the microwave-assisted fractionation of corn stover, a 250 mL of three-port flask that with 50 mL of working volume was equipped in a microwave reactor (COOLPEX). During the process, 5 g of corn stover powder was mixed-well with 50 g of the as-prepared IL, and the slurry was poured into the three-port flask. The reactions were conducted for 3 min with a stirring rate of 500 rpm, and the microwave power was in range of 130–230 W (for details of the temperature increasing curves under different microwave power, please see Additional file 1: Figure S1). After terminated the reaction, the slurry was cooling down to ~ 60 °C and the solid fraction that collected after vacuum filtration was washed by anhydrous ethanol until the pH on the surface was neutralized. After drying out at 80 °C in vacuum, the pulp was collected and stored at − 20 °C. The fractionated lignin was isolated by adding ~ 300 mL of deionized water in the liquidous fraction. The solid fraction was collected by centrifugation, followed by deionized water washing until realize the natural pH on the surface of particle. Finally, the lignin specimens were collected after freeze-drying. All the fractionation experiments were repeated in triplicate.

### Saccharification of the corn stover pulp

Batch enzymatic hydrolysis of the IL fractionated corn stover pulp was conducted to evaluate the monomeric sugars yield. Generally, 60 mg of pulp was implemented in a 2-mL tube and mixed with 1 mL of citrate buffer (50 mmol, pH 4.8). Then, 20 FPU g^−1^ (of pulp) of cellulase was added into the mixture. The enzymatic hydrolysis was conducted at 50 °C and 200 rpm for 72 h. Afterwards, the liquid fraction in hydrolysate was collected and diluted by 5 mM H_2_SO_4_ solution for further analysis. All the experiments were repeated in triplicate.

### ABE fermentation of corn stover hydrolysate

ABE fermentations were conducted using the hydrolysate of the protic IL fractionated pulps. Laboratory stored *Clostridium acetobutylicum* ABE P-1201 was adopted. The synthetic medium was used as the control group, which consisted of 40 g L^−1^ of glucose, 2.2 g L^−1^ of ammonium acetate, 1 g L^−1^ of KH_2_PO_4_, 1 g L^−1^ of K_2_HPO_4_, 0.2 g L^−1^ of MgSO_4_, 0.01 g L^−1^ of MnSO_4_, 0.01 g L^−1^ of FeSO_4_, 0.1 mg L^−1^ of biotin and 1 mg L^−1^ of *p*-aminobenzoic acid [51]. To investigate the influence of the residual [TEA][HSO_4_] in hydrolysate to the biomass growth and ABE metabolism. The synthetic medium that with similar glucose and xylose content to the corn stover hydrolysate was adopted as the substrate, in which different concentrations of [TEA][HSO_4_] were also added before the inoculation. For the ABE fermentation using the realistic corn stover hydrolysate, after pH neutralization (~ 7) by ammonium hydroxide, the obtained liquor that without additional nutrients’ supplementation was directly utilized as the medium. Batch ABE fermentation was carried out in 50 mL of anaerobic bottles that with 50 mL working volume. Before inoculation with 10 % (v/v) of the active seeds (cultivated in the synthetic medium that containing 30 g L^−1^ of glucose as carbon source), the medium was autoclaved at 115 °C for 20 min. After cooling down to the room temperature (~ 25 ^°^C), the sterilized N_2_ (> 99.99 %) was spraying into the bottle for ~ 15 min, in order to construct the anaerobic environment. Fermentation was conducted at 37 °C and 50 rpm. Samples were taken during the fermentation process for the following analysis.

### Assay

Chemical composition of the raw corn stover and the fractionated pulps were determined by the analytical procedure of NREL [52]. Sugars in enzymatic hydrolysates were analyzed by high performance liquid chromatography (Alliance® HPLC, Waters, China) that equipped with a Bio-Rad Aminex HPX-87H column and a refractive index detector. 5 mM H_2_SO_4_ solution was used as the mobile phase, and the flow rate was 0.5 mL min^−1^ [53].

The surface morphologies of the pulps were observed by scanning electron microscope (Hitachi S-3000N), and the crystallinity of cellulose was analyzed by X-ray diffractometer (XRD, Rigaku Smart Lab 9 kW, Japan). Fourier transform infrared spectrometer (FT-IR, Thermo-Fisher Nicolet 6700, US) was used to analyze the functional groups and the interlinkages of pulps and lignins. The structural information and the functional groups of the IL fractionated lignin specimens were determined by 2D-heteronuclear single quantum coherence (2D-HSQC) using fully digital superconducting NMR (AVANCE III HD 600 MHz, German) that equipped with an ultracold probe (4/7 mm solid probe). The molecule weight of the lignin specimens was measure by a high-performance gel permeation chromatography (GPC, Waters 1525, US).

### Supplementary Information

**Additional file 1** of Rapid fractionation of corn stover by microwave-assisted protic ionic liquid [TEA][HSO_4_] for fermentative acetone-butanol-ethanol production: **Figure S1.** Time courses for the temperature increases under different microwave power during the [TEA][HSO_4_] fractionation. **Figure S2.** Correlation between the corn stover delignification and the glucose yield after enzymatic hydrolysis. **Figure S3.** Correlation between the cellulose content in pulps and the glucose yield after enzymatic hydrolysis of the pulps. **Table S1.** Current advances in lignocelluloses fractionation by different types of protonic ILs. **Table S2.** Attribution of various units in the isolated lignin specimens [S10].

## Data Availability

The data that support the findings of this study are available from the corresponding author upon reasonable request.
