# Supplementary material for: Rapid fractionation of corn stover by microwave-assisted protic ionic liquid [TEA][HSO4] for fermentative acetone–butanol–ethanol production
Source: Biotechnol Biofuels Bioprod. 2024 May 7;17:62. doi: 10.1186/s13068-024-02499-0 (PMC11077788; doi:10.1186/s13068-024-02499-0)
Supplement: Supplementary file 1 — Additional file 1 of Rapid fractionation of corn stover by microwave-assisted protic ionic liquid [TEA][HSO4] for fermentative acetone-butanol-ethanol production: Figure S1. Time courses for the temperature increases under different microwave power during the [TEA][HSO4] fractionation. Figure S2. Correlation between the corn stover delignification and the glucose yield after enzymatic hydrolysis. Figure S3. Correlation between the cellulose content in pulps and the glucose yield after enzymatic hydrolysis of the pulps. Table S1. Current advances in lignocelluloses fractionation by different types of protonic ILs. Table S2. Attribution of various units in the isolated lignin specimens [S10]. [file 13068_2024_2499_MOESM1_ESM.docx]

**Additional information**

Rapid fractionation of corn stover by microwave-assisted protic ionic liquid [TEA][HSO_4_] for fermentative acetone-butanol-ethanol production

Yankun Wang ^a,b,1^, Di Cai ^a,1^, Yongjie Jiang ^a,b^, Xueying Mei ^b^, Wenqiang Ren ^c^, Mingyuan Sun ^b^, Changsheng Su ^a^, Hui Cao ^a^, Changwei Zhang ^a,^*, Peiyong Qin ^b,^*,

^a^ National Energy R&D Center for Biorefinery, Beijing University of Chemical Technology, Beijing 100029, PR China

^b^ College of Life Science and Technology, Beijing University of Chemical Technology, Beijing 100029, PR China

^c^ Research Center for Eco-environmental Sciences, Chinese Academy of Science, Beijing 100085, China

^1^ Equal contributors

* Corresponding authors

Emails:

zhangchangweibuct@163.com (C. Zhang);

qinpeiyong@tsinghua.org.cn (P. Qin)


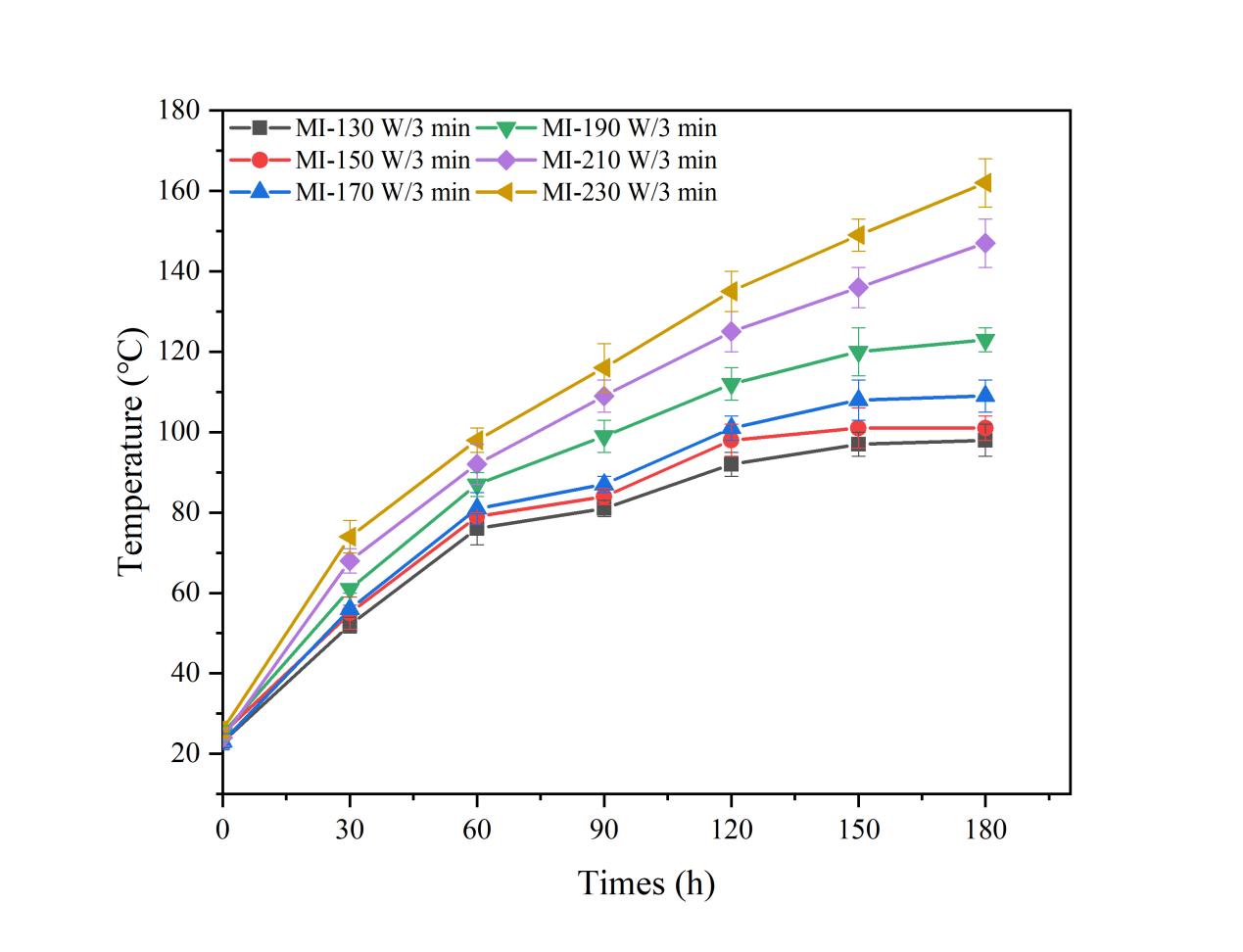


**Fig.S1** Time courses for the temperature increases under different microwave power during the [TEA][HSO_4_] fractionation.


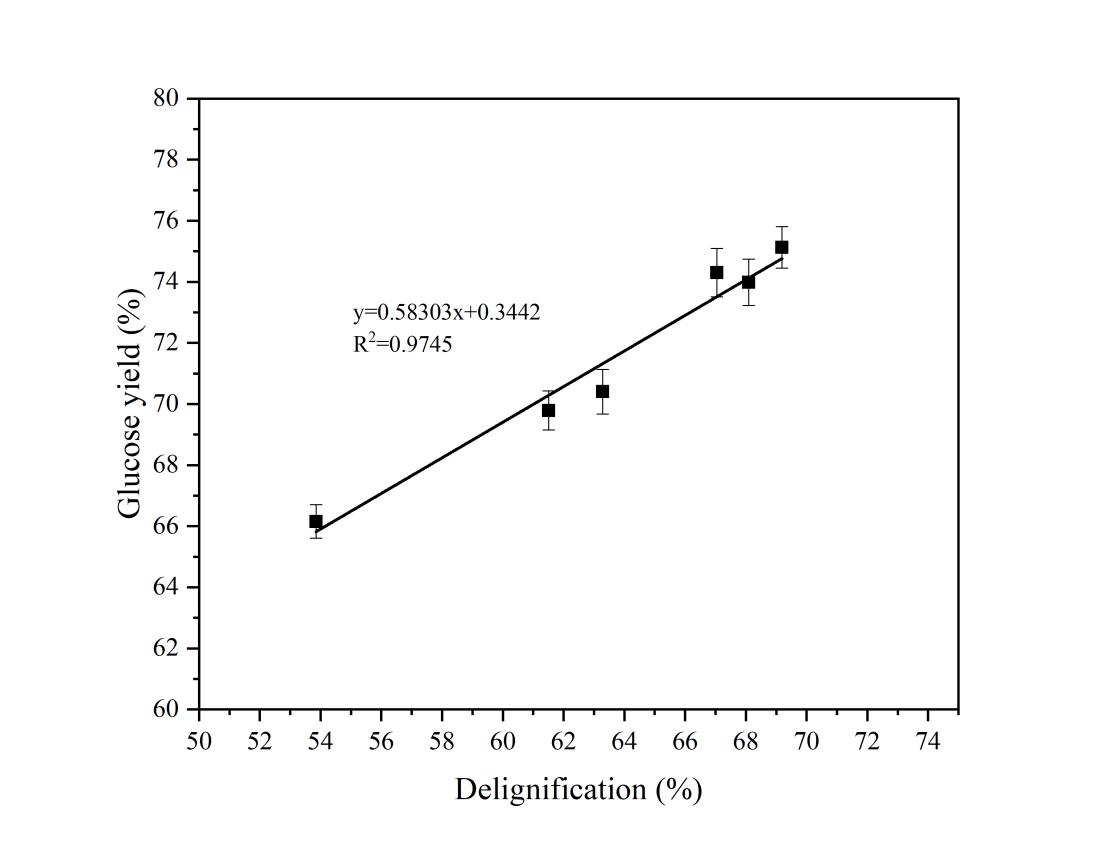


**Fig.S2** Correlation between the corn stover delignification and the glucose yield after enzymatic hydrolysis.


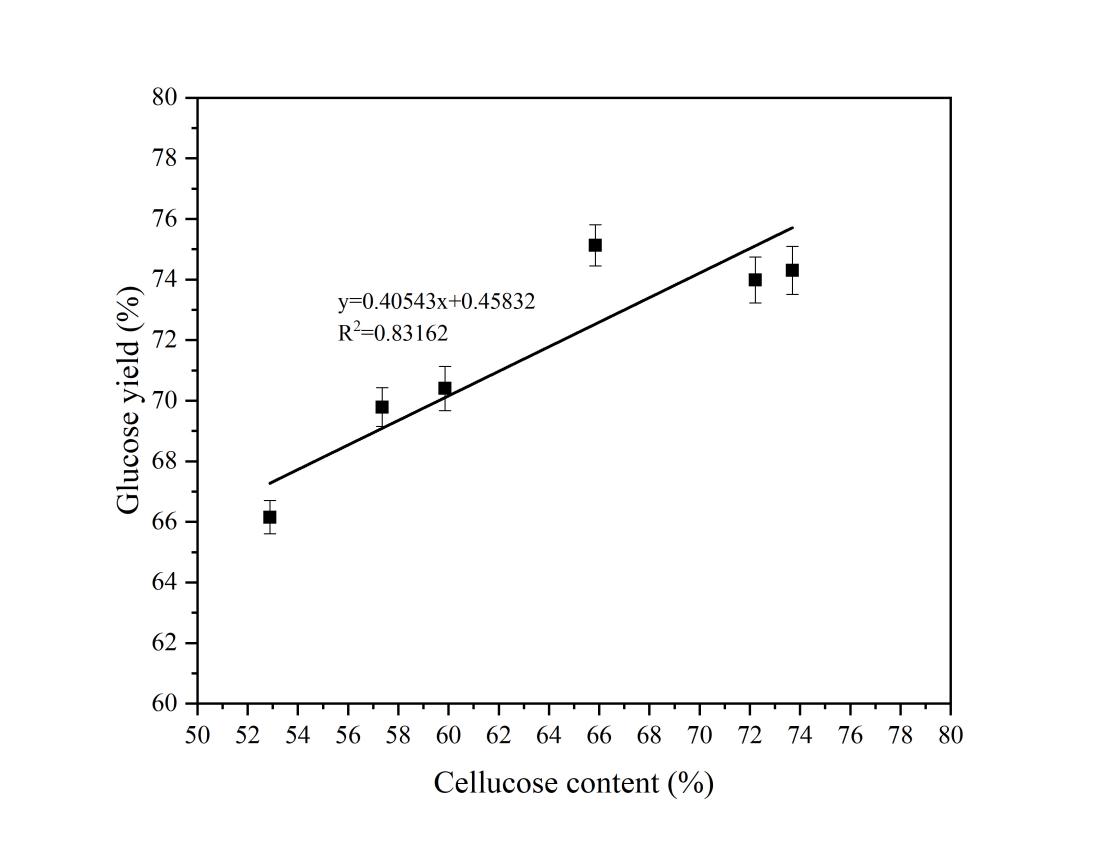


**Fig.S3** Correlation between the cellulose content in pulps and the glucose yield after enzymatic hydrolysis of the pulps.

**Table S1** Current advances in lignocelluloses fractionation by different types of protonic ILs.

| **Type of protonic ILs** | **Feedstock** | **Conditions** | **Glucose yield (%)^a^** | **Ref.** |
| --- | --- | --- | --- | --- |
| [TEA][HSO_4_] | Corn stover | MI, 190 W, 3 min | 75 | This study |
| [TEA][HSO_4_] | Corn stover | CHI, 120 ℃, 60 min | 66 | This study |
| [TEA][HSO_4_] | Sugarcane bagasse | CHI,120 ℃, 240 min | 69 | [S1] |
| [TEA][HSO_4_] | Cotton stalks | CHI,120 ℃, 240 min | 72 | [S2] |
| [TEA][HSO_4_] | Willow | CHI,150 ℃, 60 min | 82 | [S3] |
| [TEA][HSO_4_] | Miscanthus | CHI,170 ℃, 30 min | 77 | [S4] |
| [TEA][HSO_4_] | Wheat straw | CHI,170 ℃, 30 min | 81 | [S5] |
| [HBim][HSO_4_] | Pine | CHI,170 ℃, 30 min | 75 | [S4] |
| [HBim][HSO_4_] | Cotton stalks | CHI,120 ℃, 240 min | 84 | [S2] |
| [HBim][HSO_4_] | Miscanthus giganteus | CHI,120 ℃, 1440 min | 80 | [S6] |
| [DMBA][HSO_4_] | Husk | CHI,170℃, 45 min | 82 | [S7] |
| [EOA][OAc] | Almond | CHI,160 ℃,180 min | 82 | [S8] |

^a^$G\mathrm{lucose} \mathrm{yield}(\%) =\frac{C_{g}\times0.9}{C_{d}}\times100$%, where $C_{g}$ is the weight of glucose after enzymatic hydrolysis;$\mathrm{and}C_{d}$ is glucan content in the pulp [S9].

**Table S2** Attribution of various units in the isolated lignin specimens [S10].

| **Label** | ***δ_C_* (ppm)** | ***δ_H_* (ppm)** | **Assignment** |
| --- | --- | --- | --- |
| *B’_β_* | 49.8 | 2.56 | *C_β_-H_β_* in *β-β* tetrahydrofuran (B’) |
| *C_β_* | 53.1 | 3.46 | *C_β_-H_β_* in phenylcoumaran (C) |
| *B_β_* | 53.4 | 3.07 | *C_β_-H_β_* in *β-β* (resinol) (B) |
| *OCH_3_* | 56.3 | 2.75 | *C-H* in methoxyl |
| *A_γ_* | 59.8 | 3.35-3.80 | *C_γ_-H_γ_* in *β-O-4* substructures (A) |
| *A’_γ_* | 63.2 | 4.36 | *C_γ_-H_γ_* in γ-acylated *β-O-4* (A’) |
| *C_γ_* | 62.1 | 3.76 | *C_γ_-H_γ_* in phenylcoumaran (C) |
| *B_γ_* | 71.1 | 3.82-4.18 | *C_γ_-H_γ_* in *β-β* resinol (B) |
| *A_α_* | 71.8 | 4.86 | *C_α_-H_α_* in *β-O-4* unit (A) (*Eythro*) |
| *A_α_* | 71.8 | 4.86 | *C_α_-H_α_* in *β-O-4* unit (A) (*Thero*) |
| *A_β_(G)* | 83.4 | 4.38 | *C_β_-H_β_* in *β-O-4* linked to G (A) |
| *B_α_* | 84.8 | 4.66 | *C_α_-H_α_* in *β-β* resinol (B) |
| *A_β_(S)* | 85.8 | 4.12 | *C_β_-H_β_* in *β-O-4* linked to S (A, *Erythro*) |
| *A_β_(S)* | 86.7 | 4.01 | *C_β_-H_β_* in *β-O-4* linked to S (A, *Thero*) |
| *D_α_* | 81.1 | 5.11 | *C_α_-H_α_* in spirodienones (D) |
| *C_α_* | 86.8 | 5.45 | *C_α_-H_α_* in phenylcoumaran (C) |
| *T_6_* | 98.9 | 5.45 | *C_2,6_-H_2,6_* in tricin (T) |
| *T_8_* | 94.2 | 6.61 | *C_8_-H_8_* in tricin (T) |
| *S_2,6_* | 103.9 | 6.71 | *C_2,6_-H_2,6_* in syringyl units (S) |
| *G_2_* | 110.8 | 6.97 | *C_2_-H_2_* in guaiacyl units (G) |
| *G_5_* | 114.5 | 6.70 | *C_5_-H_5_* in guaiacyl units (G) |
| *G_6_* | 119.1 | 6.78 | *C_6_-H_6_* in guaiacyl units (G) |
| *H_2,6_* | 127.7 | 7.17 | *C_2,6_-H_2,6_* in H units (H) |
| *PCE_2,6_* | 130.1 | 7.48 | *C_2,6_-H_2,6_* in *p*-coumarate (*p*-CE) |
| *PCE_7_* | 144.8 | 7.51 | *C_7_-H_7_* in *p*-coumarate (*p*-CE) |
| *PCE_8_* | 113.7 | 6.24 | *C_8_-H_8_* in *p*-coumarate (*p*-CE) |
| *FA_2_* | 110.7 | 7.35 | *C_2_-H_2_* in ferulate (*p*-CE) |
| *FA_6_* | 123.1 | 7.21 | *C_6_-H_6_* in ferulate (*p*-CE) |
| *FA_7_* | 144.8 | 7.51 | *C_7_-H_7_* in ferulate (*p*-CE) |

**References**

[S1] C. L. Chambon, T. Y. Mkhize, P. Reddy, A. Brandt-Talbot, N. Deenadayalu, P. S. Fennell,J. P. Hallett, Pretreatment of South African sugarcane bagasse using a low-cost protic ionic liquid: a comparison of whole, depithed, fibrous and pith bagasse fractions, Biotechnology for Biofuels. 2018; 11(1): 247. <https://doi.org/10.1186/s13068-018-1247-0>.

[S2] I. Semerci,F. Güler, Protic ionic liquids as effective agents for pretreatment of cotton stalks at high biomass loading, Industrial Crops and Products. 2018; 125: 588-95. https://doi.org/10.1016/j.indcrop.2018.09.046.

[S3] L. Weigand, S. Mostame, A. Brandt-Talbot, T. Welton,J. P. Hallett, Effect of pretreatment severity on the cellulose and lignin isolated from Salix using ionoSolv pretreatment, Faraday Discuss. 2017; 202: 331-49. <https://doi.org/10.1039/c7fd00059f>.

[S4] F. J. V. Gschwend, C. L. Chambon, M. Biedka, A. Brandt-Talbot, P. S. Fennell,J. P. Hallett, Quantitative glucose release from softwood after pretreatment with low-cost ionic liquids, Green Chemistry. 2019; 21(3): 692-703. <https://doi.org/10.1039/c8gc02155d>.

[S5] C. L. Chambon, M. Chen, P. S. Fennell,J. P. Hallett, Efficient fractionation of lignin- and ash-rich agricultural residues following treatment with a low-cost protic ionic liquid, Front Chem. 2019; 7: 246. <https://doi.org/10.3389/fchem.2019.00246>.

[S6] P. Verdía, A. Brandt, J. P. Hallett, M. J. Ray,T. Welton, Fractionation of lignocellulosic biomass with the ionic liquid 1-butylimidazolium hydrogen sulfate, Green Chemistry. 2014; 16(3). <https://doi.org/10.1039/c3gc41742e>.

[S7] S. O. Anuchi, K. L. S. Campbell,J. P. Hallett, Effective pretreatment of lignin-rich coconut wastes using a low-cost ionic liquid, Scientific Reports. 2022; 12(1): 6108. <https://doi.org/10.1038/s41598-022-09629-4>.

[S8] L. Das, E. C. Achinivu, C. A. Barcelos, E. Sundstrom, B. Amer, E. E. K. Baidoo, B. A. Simmons, N. Sun,J. M. Gladden, Deconstruction of woody biomass via protic and aprotic ionic liquid pretreatment for ethanol production, ACS Sustainable Chemistry & Engineering. 2021; 9(12): 4422-32. <https://doi.org/10.1021/acssuschemeng.0c07925>.

[S9] D. Fu,G. Mazza, Aqueous ionic liquid pretreatment of straw, Bioresource Technology. 2011; 102(13): 7008-11. [https://doi.org/10.1016/j.biortech.2011.04.049](https://doi.org/https://doi.org/10.1016/j.biortech.2011.04.049).

[S10] J.-L. Wen, S.-L. Sun, B.-L. Xue,R.-C. Sun, Quantitative structural characterization of the lignins from the stem and pith of bamboo (*Phyllostachys pubescens*), 2013; 67(6): 613-27. <https://doi.org/doi:10.1515/hf-2012-0162>.
